# Supplementary material for: Perceptions around COVID-19 and vaccine hesitancy: A qualitative study in Kaski district, Western Nepal
Source: PLOS Glob Public Health. 2023 Feb 17;3(2):e0000564. doi: 10.1371/journal.pgph.0000564 (PMC10022296; doi:10.1371/journal.pgph.0000564)
Supplement: S1 File — (DOCX) [file pgph.0000564.s002.docx]

**अनुसन्धान सुसूचित मन्जुरीनामा फारम**

यो अध्ययनको उदेश्य “कास्की जिल्लामा कोभिड बिरुद्दमा लगाइएको खोपसंग सम्बन्धि सहजता तथा अवरोधहरूको बारेमा तपाईंको विचार र दृष्टिकोण” जान्ने हो l यस अनुसन्धानले तपाइलाई व्यक्तिगत रुपमा कुनै पनि हानी नोक्सानी पुर्याउने छैन र यसमा सोधिएका प्रश्न तपाईले निर्धक्क भई जवाफ दिन सक्नुहुनेछ किनकी ति जवाफहरु तपाई र अनुसन्धान कर्ता विच पूर्ण रुपमा गोप्य रहनेछ l यस क्रममा तपाई कुनै पनि बेला अनुसन्धान प्रकृयाबाट बाहिरिन खोजेमा तपाईको निर्णयको सम्मान गरिने छ l

हामी तपाईंलाई प्रश्नहरू सोध्नेछौं, जुन २० देखि ३० मिनेट लाग्न सक्छ र यो हामी रेकोर्ड गर्छौ , यसमा हजुर सहमत हुनुहुन्छ नि l .......धन्यवाद , अब हामी प्रश्न तिर लागौ है त l

नाम:

उमेर:

लिङ्ग:

शैक्षिक योग्यता:

पेशा :

अस्थायी बसोबास :

वाड नम्बर :

**कोरोना महामारी सम्बन्धि :**

1. कोभिड महामारीको बारेमा हजुरको धारणा के छ ? यसले तपाई र तपाईंको परिवारमा कस्तो असर पारिराखेको छ ?(प्रोब : शारीरिक ,मानसिक आर्थिक )
2. के तपाईं वा तपाईंको परिवारमा अथवा आफन्तमा कोही कोरोना भाइरस संक्रमित हुनुहुन्थ्यो/ छ ? यदि हुनुहुन्छ भने , संक्रमण कतिको गम्भीर थियो , कस्तो अवस्था थियो/छ ?
3. के तपाईंलाई कोरोना भाइरस धेरै खतरनाक संक्रमण हो जस्तो लाग्छ? (प्रोब : तपाईंलाई किन त्यस्तो लाग्छ ?)

**कोरोना खोप सम्बन्धि :**

1. अहिले जुन कोरोना बिरुद्धमा खोप आएको छ , त्यसको बारेमा केहि बताइदिनुस l
2. कोरोनाभाइरसको लागि कुनै प्रभावकारी उपचार नभए पनि संक्रमणलाई रोक्न बैज्ञानिकहरुले खोपको विकास गरेका छन् l यी खोपहरु धेरै देशमा लगाउन सुरु भइसकेको छ, तपाईंको विचारमा के यो खोप सबैले लगाउनाले यो महामारी अन्त्य हुन्छ त ?
3. तपाईं आफैंले वा तपाइको परिवारको कुनै सदस्यले खोप लगाउनुभयो ? यदि छ भने तपाईलाई यो खोप किन लगाउनुपर्छ जस्तो लाग्यो ?

यदि छैन भने, भविष्यमा आगामी दिनमा लगाउन चाहनुहुन्छ ? किन ?

1. तपाइको बिचारमा कोभिड खोपको सबल र नकारात्मक पक्ष के के हुन् ?
2. के तपाईं कोभिड विरुद्धको खोपको कुनै पक्षसँग चिन्तित हुनुहुन्छ ? तपाईंलाई के लाग्छ ? (उदाहरण रगत जम्ने , नकारात्मक असर , खोपको composition ,गर्भवती सुत्केरी अवस्थामा लिने कि नलिने अन्योल, प्रजनन क्षमतामा असर पर्ने डर)
3. मानिसहरु विभिन्न कारणले खोप लिन चाहन्नन् (माथि उल्लेख गरे जस्तै ) तपाईंलाई के लाग्छ, उनीहरुलाई खोप लिन कसरी मनाउन सकिन्छ ?
