# Supplementary material for: Perceptions around COVID-19 and vaccine hesitancy: A qualitative study in Kaski district, Western Nepal
Source: PLOS Glob Public Health. 2023 Feb 17;3(2):e0000564. doi: 10.1371/journal.pgph.0000564 (PMC10022296; doi:10.1371/journal.pgph.0000564)
Supplement: S2 File — (DOCX) [file pgph.0000564.s003.docx]

S2 Interview Guide

| 1. **Socio-demographic Variables** |
| --- |
| Name of participants/Code no |
| Age |
| Sex |
| Occupation |
| Place of residence |
| Ward no |
| Status of COVID vaccine |
| Education Level |
| Religion |
| 1. **Questions related to COVID** |
| 1. Lead question: First tell me how have you been throughout this pandemic?  Probe question: How has it affected you and your family members and in what ways: physical, mental, financial, etc? |
| 2.Lead: Have you or any of your family members or relatives been infected with coronavirus?  Probe: If yes, how severe was the infection and if they recovered or died? |
| 3.Lead: Do you think Coronavirus is very dangerous infection?  Probe: Why do you think so? |
| **C.COVID -Vaccine related Questions:** |
| 1. Tell me what do you know about the COVID vaccine? |
| 2. Since there is no effective treatment for this but to prevent the infection scientist have developed vaccines. This vaccine has already been rolled out in several countries, do you think providing vaccines to majority of people in every country in the world will help end the pandemic? |
| 3.Lead: Have you yourself or any of your family member had the vaccine?  Probe: If yes, why did you had it, if no do you want to get it in future and why? |
| 4. Lead: In your opinion, what are the pros and cons of uptaking of COVID vaccine? |
| 5. Lead: Are you concerned with any aspect related to COVID vaccines, what do you think could be the reasons? (for example safety - reports of blood clots found in some people, side effects after having it, concern about composition of vaccine, concern about whether to take while pregnant, breastfeeding or if it affects fertility etc) |
| 6. Lead: Some people don’t want to take vaccines for several reasons (as mentioned above).  Probe: How do you think they can be persuaded to take the vaccine? |
