# Supplementary material for: Perceptions around COVID-19 and vaccine hesitancy: A qualitative study in Kaski district, Western Nepal
Source: PLOS Glob Public Health. 2023 Feb 17;3(2):e0000564. doi: 10.1371/journal.pgph.0000564 (PMC10022296; doi:10.1371/journal.pgph.0000564)
Supplement: Attachment — Submitted filename: RESPONSE TO REVIEWERS+EvT.docx [file pgph.0000564.s005.docx]

Dear Editor

PGPH-D-22-00044 ‘Perceptions around COVID-19 and vaccine hesitancy: A qualitative study in Kaski district, Western Nepal’

Thank you for the reviewers’ comments & suggestions. We have addressed each point in turn below:

- It is not clearly explained who the participants were. It is stated that "Details of participants were obtained from health facility mangers" but it's not clear how these managers selected them. Please provide clear inclusion criteria and describe how participants were identified. Remove duplication of material regarding identification of participants from the bottom of pages 9 and 10.

Authors’ reply: We have tried best to clear this in the Materials and Methods under study settings sub-heading.

- You state that "The number of participants in this study was deemed adequate based on the principles of data saturation whereby subsequent interviews did not yield new information". Do you mean that you conducted interviews until you reached saturation?

Authors’ reply: Yes, we meant that the interviews were conducted until we reached saturation, this has now clearly been mentioned in the text.

- Please provide dates when interviews took place. This is crucial context given the changing dynamics of the pandemic.

Authors’ reply: We have now provided the month and year when the interviews were done

Please shorten and refocus introduction. The first paragraph of the section "Role of vaccines in preventing morbidity and mortality" can be removed.

Authors’ reply: Thank you. we have removed the section ‘ Role of vaccines in preventing morbidity and mortality’.

- Keep material relevant to the research topic. For example we don't need to know too much detail about how Nepal's health system is structured, but it would be useful to know about the course of pandemic - cases/ deaths relative to other countries, vaccination rate - up to the point when interviews took place.

Authors’ reply: Thank you very much for the suggestion. Appreciate this suggestion. We have revised it based on your suggestion and reads as follows:

Nepal tackled COVID-19 pandemic amidst the ongoing transformation in health system and corresponding challenges [8, 13]. Nepal detected a total of 979, 607 cases, and almost 12,000 lost lives due to COVID-19. Almost 68% of population has received completed two doses of COVID-19 vaccines with 9% partially vaccinated, against the backdrop of grim vaccination rate (17.8%) in low and middle income countries (LMICs) [14]. One in third of Nepali population is still unvaccinated and some of the reasons behind poor vaccination are inherent in perceptions around COVID-19 and the vaccine hesitancy. The main objective of this study was to explore perceptions around COVID-19 and vaccine hesitancy among urban and rural population in Kaski district of Western Nepal.

- What language/s were interviews conducted in?

Authors’ reply: Interviews were conducted in Nepali language, this is mentioned in the manuscript (please see page 7 second line).

- Why was Kaski district selected?

Authors’ reply: Kaski was selected as it is offers a good mix of rural and urban residents, it contains the second largest city of Nepal as well as contains rural areas.

- Please reduce the existing discussion and add material on how your findings compare to findings from other countries?

Authors’ reply: Thank you for the suggestion. We have revised the discussion to reduce it wherever possible and added the comparative literature that adds to our findings.

- You should include 1-2 paragraphs regarding study limitations. e.g. how generalisable are the findings to Nepal's population?

Authors’ reply: We have now provided one paragraph on strength and limitations of the study

- Please provide the interview guide/schedule. If it fits in a table/ box include in main manuscript, otherwise as supplemental material.

Authors’ reply: Thank you for the suggestion. We have included it in the supplementary file.

- Please add a box/ table with a summary of recommendations for policy makers/ health services on how to increase vaccine uptake.

Authors’ reply: We have provided a box with a summary of our recommendations

- First/last/corresponding authors are affiliated in UK. You may want to mention that first author completed undergraduate studies in Nepal.

Authors’ reply: We have briefly mentioned the study and work experience of the first author in Nepal.

We have also added a recent 2022 reference, we have renamed the Background section to ‘Introduction’ (as requested), we have move Table 1 into manuscript (it was separate file in initial submission), we have edited final disclosure section as requested.

Yours sincerely,

Prof. Edwin van Teijlingen on behalf of my co-authors.
